# Correlation analysis of cold-related gene expression with physiological and biochemical indicators under cold stress in oil palm

**DOI:** 10.1371/journal.pone.0225768

**Published:** 2019-11-27

**Authors:** Jing Li, Yaodong Yang, Amjad Iqbal, Rashad Qadri, Peng Shi, Yong Wang, Yi Wu, Haikuo Fan, Guojiang Wu

**Affiliations:** 1 Hainan Key Laboratory of Tropical Oil Crops Biology/Coconut Research Institute, Chinese Academy of Tropical Agricultural Sciences, Wenchang, Hainan, China; 2 Key Laboratory of Plant Resource Conservation and Sustainable Utilization, South China Botanical Garden, Chinese Academy of Sciences, Guangzhou, China; National Institute of Plant Genome Research, INDIA

## Abstract

Oil palm (*Elaeis guineensis* Jacq.) is a representative tropical oil crop that is sensitive to low temperature. Oil palm can experience cold damage when exposed to low temperatures for a long period. During these unfavorable conditions, a series of gene induction/repression and physico-chemical changes occur in oil palm. To better understand the link between these events, we investigated the expression levels of various genes (including *COR410*, *COR413*, *CBF1*, *CBF2*, *CBF3*, *ICE1-1*, *ICE1-2*, *ICE1-4*, *SIZ1-1*, *SIZ1-2*, *ZAT10*, *ZAT12*) and the accumulation of osmolytes (proline, malondialdehyde and sucrose). Likewise, the activity of superoxide dismutase (SOD) in oil palm under cold stress (4°C, 8°C and 12°C) was examined. The results showed a clear link among the expression of *CBFs* (especially *CBF1* and *CBF3*) and the all genes examined under cold stress (12°C). The expression of *CBF1* and *CBF2* also exhibited a positive link with the accumulation of sucrose and proline under cold stress in oil palm. At 4°C, the proline content exhibited a very significant correlation with electrolyte leakage in oil palm. The results of this study provide necessary information regarding the mechanism of the response and adaption of oil palm to cold stress. Additionally, they offer clues for the selection or development of cold-tolerant cultivars from the available germplasms of oil palm.

## Introduction

Oil palm (*Elaeis guineensis* Jacq.) is a representative tropical oil crop that belongs to the family Arecaceae and genus *Elaeis*, which contains two species, *E*. *guineensis* (African oil palm) and *E*. *oleifera* (American oil palm). At present, most oil palm is cultivated in Southeast Asia, Africa, the Brazilian tropics and Central America. It is the most productive oil crop in the world, yielding an average of 4.27 tons of vegetable oil per hectare per year. Oil palm accounts for only 5% of the world’s oil crop cultivation area and supplies approximately 33% of vegetable oil and 45% of edible oil used worldwide [1]. The global demands for vegetable oil have greatly increased over the years in terms of consumption due to the rapid expansion of the population. Such soaring trends in population and consumption could represent a constant threat to sustainable oil production. However, this threat can be addressed by proper planning or management of oil palm production [2].

The optimum growth conditions for oil palm are temperatures of 24 to 28°C [3] with evenly distributed average annual rainfall of 2000–3000 mm. The minimal growth temperature for oil palm is 15°C [4, 5], and the optimum mean temperature is approximately 27°C [6]. Generally, the oil palm plantation extends over a geographical range of 10° N to 10° S [6]. However, low temperatures below 20°C can limit the growth of oil palm and even halt it at 12°C [7]. Such low temperatures in the subtropics during winter can negatively affect oil palm growth, fruit development and, consequently, oil production.

As plants sense cold stress, a series of physico-chemical indicators are induced at the molecular and cellular levels to protect plants from cold damage (Browse, 2001). Most temperate plant species have evolved specialized mechanisms at the physiological, molecular and biochemical levels to survive under cold stress [8, 9]. The cold stress trigger the formation of ROS which degrade polyunsaturated lipids to form MDA, a reactive aldehyde that initiates toxic stress in cells and subsequently causes cellular dysfunction and tissue damage [10]. The formation of ROS also induces the expression of the SOD gene to increase the total SOD activity. Accumulation of cryoprotectants, like soluble sugars that accumulate in plants under stress include sucrose, hexose, raffinose, glucose, fructose, and trehalose. These sugars act as compatible solutes in freezing stress, serving as osmoprotectants against freezing-dehydration damage [11, 12]. Proline, another cryoprotectants accumulation is also enhanced by cold stress. In addition to acting as a reservoir of carbon and nitrogen, proline also protects cellular enzymes from denaturation [13].

During cold acclimation, C-repeat binding factors (CBFs) activate cold-responsive (*COR*) genes and subsequent accumulation of cryoprotectants which results in the acquisition of freezing tolerance [14]. During the cold stress, the gene expression profile was altered and among them *CBFs* were the most studied genes. It has been shown that the expression of *CBF1* and *CBF3* during cold acclimation precedes that of *CBF2* during the cold stress at 4°C in *Arabidopsis*, and the CBF proteins may have different functional activities [15]. The three CBFs do not have fully overlapping functions. CBF1 and CBF3 play a different role than CBF2 in both constitutive freezing tolerance and cold acclimation [16]. CBF2 protein is involved in feedback regulation of CBF1 and CBF3 expression during cold acclimation. Inducer of CBF expression 1 (ICE1) is an MYC-like basic helix–loop–helix transcription factor that binds to the MYC cis-acting elements in the *CBF* promoter it is also induced under cols stress [17, 18]. SIZ1-dependent sumoylation of ICE1 may activate and/or stabilize the protein and facilitating leading to low temperature tolerance [19]. ZAT10 and ZAT12 may involve in the regulation of *CBF* independent *COR* genes expression [20]. There are genes and/or transcription factors that respond to cold stress in various species. The identified genes or transcription factors include *CBF* in *Arabidopsis*, *WRKY* in oil palm [21] and *DaCBF4* in *Deschampsia antarctica* [22].

Knowledge of *COR* (cold-responsive) gene expression followed by the transformation of oil palm with these cold tolerance genes would lead to the development of tolerant *Elaeis guineensis*. In addition to the molecular basis of the response to cold, the mechanism is also associated with a series of biochemical and physiological alterations. In the present work, the expression of 12 cold-inducible genes and the accumulation of several cold-related physiological indices of oil palm were investigated. The expression patterns of cold-inducible genes and the levels of several corresponding physiological indices were determined by Real-time RT-PCR and by using an ultraviolet spectrophotometer. Finally, the data were analyzed to identify gene expression patterns and their regulation of downstream physiological indices of cold in oil palm.

## Materials and methods

### 1. Plant materials and treatment conditions

Seedlings of the oil palm sub-species Dura (thick-shelled African oil palm) were grown in the nursery at the Hainan Key Laboratory of Tropical Oil Crops Biology/Coconut Research Institute, Chinese Academy of Tropical Agricultural Science, Wenchang, Hainan, China. Approximately twenty-four plants (12 months old) that were germinated at the same time under the same conditions were subjected to cold treatment. Before exposure to the cold treatment, the plants were placed in a growth chamber at 26°C for two days for acclimatization. The plants were then divided into four groups; one group was kept at 26°C as a control. The remaining three groups were subjected to low temperatures (4°C, 8°C, and 12°C) in a preset growth chamber under a similar photoperiod for 8 days. Spear-shaped leaves (the young leave was the leave which is 4-5th leave next to the center spear leave (unopen leave) and the mature leave was the leave that complete open and is 9-10^th^ leave from the spear leave) from the treated oil palms were picked at various time points (1 h, 7 h, 1 d, 2 d, 4 d, 6 d, 8 d) in triplicate. The sampled leaves were then processed for the physiological experiments as well as RNA extraction. All samples were immediately snap frozen in liquid nitrogen after collection and stored at -80°C for RNA extraction.

## 2. Extraction and determination of physiological data in the samples

### 2.1 Determination of Relative Electrolyte leakage (EL)

Briefly, leaf samples were cut into small pieces, and approximately 0.2 grams of each leaf sample was washed with tap water to free it of dirt. The washed pieces were then transferred to a bottle containing 40 mL of distilled water and left at room temperature for 10 h. After 10 h of incubation, the initial (S1) conductance (μs.cm^-1^) was measured with a conductance apparatus (DDS-11A). The samples were then boiled for 15 min to induce maximum leakage. After cooling at room temperature for 2 h, electrolyte conductivity (μs.cm^-1^) was measured again and recorded as S2. The final conductance was calculated using the following formula:

Conductance=S1S2×100

### 2.2 Determination of proline content

Proline contents in oil palm leaves were estimated by using the method of Shan et al. [23], but with some modifications. Approximately 0.5 g of leaf tissue was ground in liquid nitrogen and homogenized with 5 mL of 3% sulfosalicylic acid in a boiling water bath for 10 min. Subsequently, 2 mL of the aqueous extract was incubated with 2 mL of acidified ninhydrin reagent [1.25 g ninhydrin reagent dissolved in 30 mL of glacial acetic acid and 20 mL of 6 mol/L phosphoric acid] and 2 mL of glacial acetic acid in a boiling water bath for 30 min. After cooling, the reaction mixture was partitioned against toluene (5 mL), and the absorbance of the organic phase was measured at 520 nm using UV spectrophotometry. The proline contents of the leaves were calculated from the standard curve constructed by using known amounts of proline (Sigma, St Louis, MO, USA).

### 2.3 Determination of Malondialdehyde (MDA) content

The MDA contents of the oil palm leaves were estimated according to the method described by Feng et al., [24]. Approximately 0.5 g of leaf tissue was homogenized in 5 mL of 10% (w/v) trichloroacetic acid (TCA), and the homogenate was transferred to a Greiner tube, which was then centrifuged at 10,000 × g for 10 min at room temperature. The supernatant (2 mL) was transferred to a new tube and mixed with an equal volume of thiobarbituric acid (TBA, 0.6% in 10% TCA). The mixture was boiled at 100°C for 15 min and immediately chilled on ice. After chilling, the contents of the tube were further centrifuged for 10 min at 4000 × g to clarify the solution. Finally, the absorbance of the supernatant was measured at 532 nm and 600 nm. The final absorbance was recorded by subtracting the absorbance reading at 600 nm from that at 523 nm to avoid the effect of nonspecific turbidity.

### 2.4 Determination of Superoxide dismutase (SOD)

The activity of the antioxidant enzyme superoxide dismutase (SOD) was estimated using a Total SOD Assay Kit (A001-1; Nanjing Jiancheng, Nanjing city, China), as described by Sun [25]. And 35mL supernatant was used for determinating the SOD activity.

### 2.5 Determination of sucrose

The sucrose content was estimated according to the protocol described by Rutten et al[11].

## 3. RNA isolation and cDNA synthesis

Total RNA was isolated from the control and cold-treated leaf samples using the MRIP method described by Xiao et al. [26], and the quality was verified by gel electrophoresis.

First-strand cDNA was synthesized using the RevertAid^TM^ First Strand cDNA Synthesis Kit (Fermentas, Lithuania) according to the manufacturer’s instructions, and the concentration was then adjusted to 50 ng/μL using an ND-1000 UV-Vis spectrophotometer (NanoDrop, USA).

## 4. Real-time quantitative PCR

Gene expression changes were determined according to a standard SYBR Premix EX Taq Kit protocol in 384-well optical plates (Axygen). The Applied Biosystems 9700 Real-time RT-PCR System (Applied Biosystems, USA) was used with the following conditions: 50°C for 2 min, 95°C for 2 min, followed by 40 cycles at 95°C for 15 s and 60°C for 1 min, then 95°C for 15 s, 60°C for 15 s and 95°C for 15 s. Each 10 μL reaction mixture contained 5 μL of 2 × SYBR GREEN PCR Master Mix (TOYOBO, Japan), 0.5 μL of each forward and reverse primer at 10 μM, 1 μL of cDNA template and 3 μL of ddH_2_O.

Primers for Real-time RT-PCR analysis were designed with Primer Express software (Applied Biosystems, USA) and synthesized at the Beijing Genomics Institute (BGI) (Guangzhou, China) as shown in S1 Table. To normalize the amount of cDNA template, the housekeeping gene Eg-actin was amplified along with the target genes as an endogenous control using the forward primer 5’-GTTGTCGCTCCACCCG-3’ and the reverse primer 5’-GCAGGACCACATTCATCATA -3’. All primers were validated for correct amplification by cloning and sequencing the PCR products.

## 5. Data analysis

Data were analyzed and evaluated by using SAS software (SAS Inc., Cary, NC, USA) with the CANCORR and GLM procedures, and the results are shown as the mean ± standard error (n = 4 for Real-time RT-PCR, and n = 3 for physiological data evaluation).

## Results

### Expression of cold-inducible genes under cold stress

**Fig 1:**
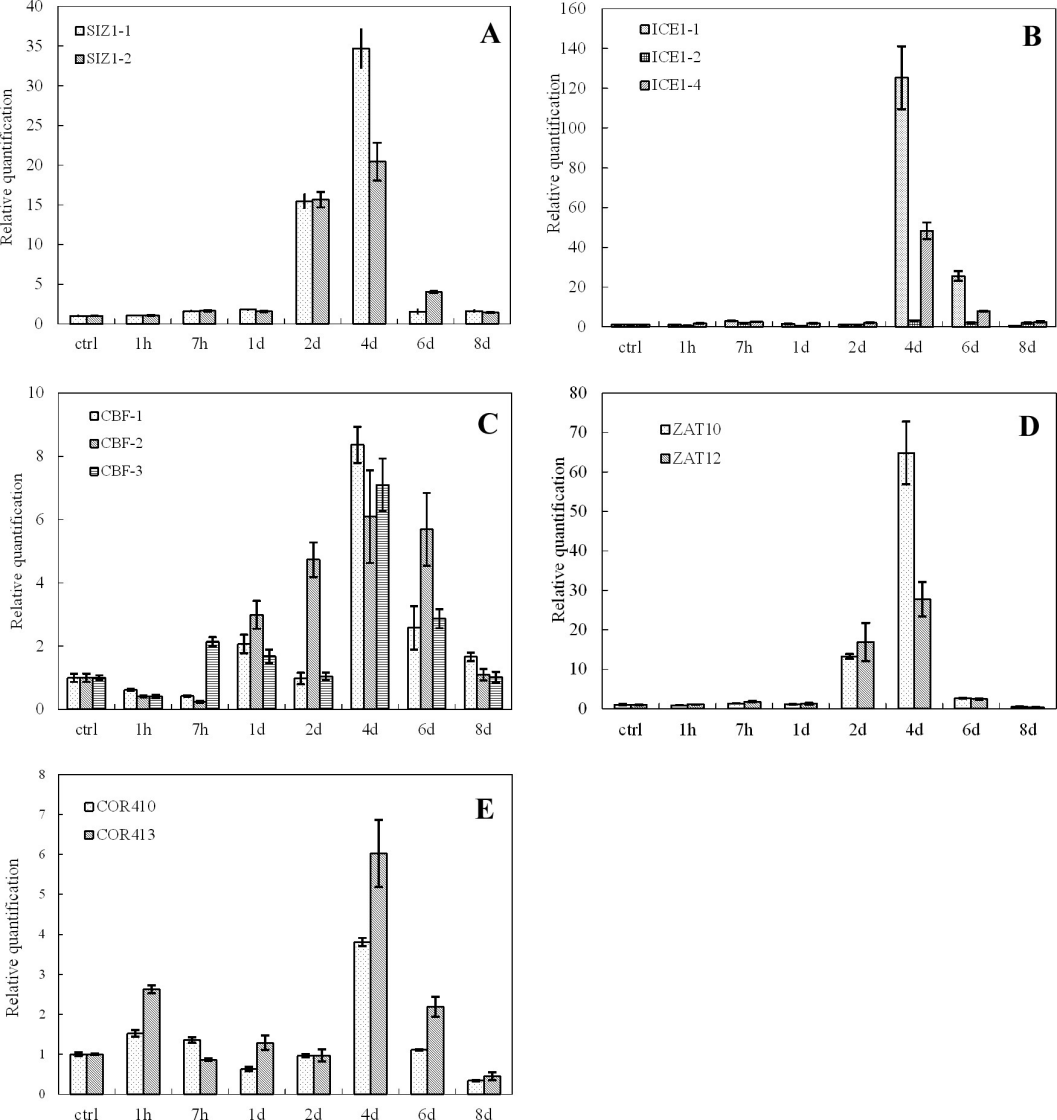
Expression of cold-inducible genes in oil palm under cold stress (4°C). A. *SIZ1-*1 and *SIZ1-*2; B. *ICE1*-1, *ICE1*-2 and *ICE1*-4; C. *CBF1*.*CBF2* and *CBF3*; D. *ZAT10* and *ZAT12*; E. *COR410* and *COR413*. Different lower case letters (a, b, c, d, e) above the bars indicates significant differences between genes expression at different time point (*p* <0.05), correspondingly. a is significantly higher than b, c and d, b is significantly higher than c and d and so on. (on x-axis ctrl = control, h = hour, d = day).

**Fig 2:**
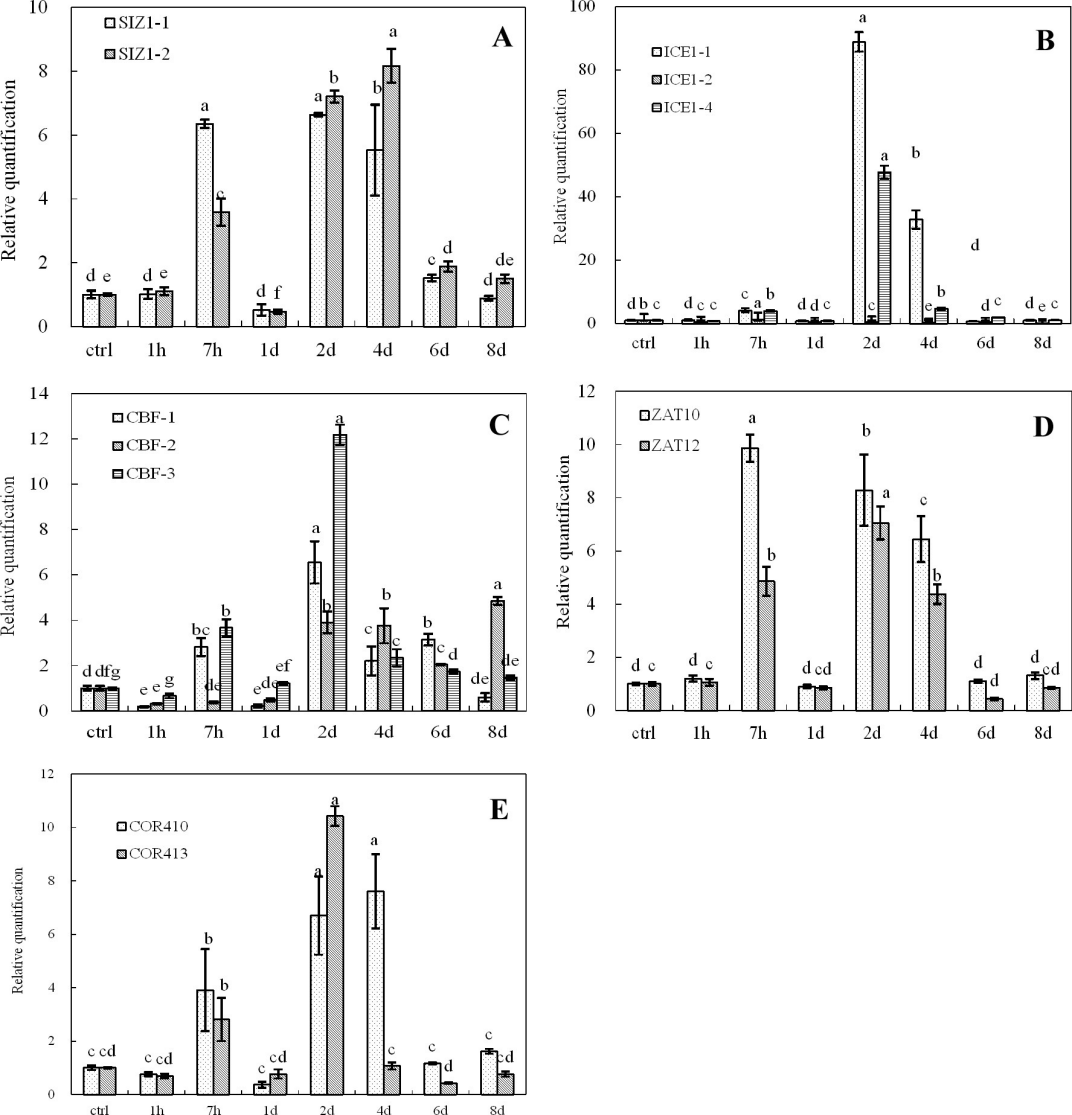
Expression of cold-inducible genes in oil palm under cold stress (8°C). A. *SIZ1*-1 and *SIZ1*-2; B. *ICE1*-1, *ICE1*-2 and *ICE1*-4; C. *CBF1*.*CBF2* and *CBF3*; D. *ZAT10* and *ZAT12*; E. *COR410* and *COR413*. Different lower case letters (a, b, c, d, e) above the bars indicates significant differences between genes expression at different time point (*p* <0.05), correspondingly. a is significantly higher than b, c and d, b is significantly higher than c and d and so on. (on x-axis ctrl = control, h = hour, d = day).

**Fig 3:**
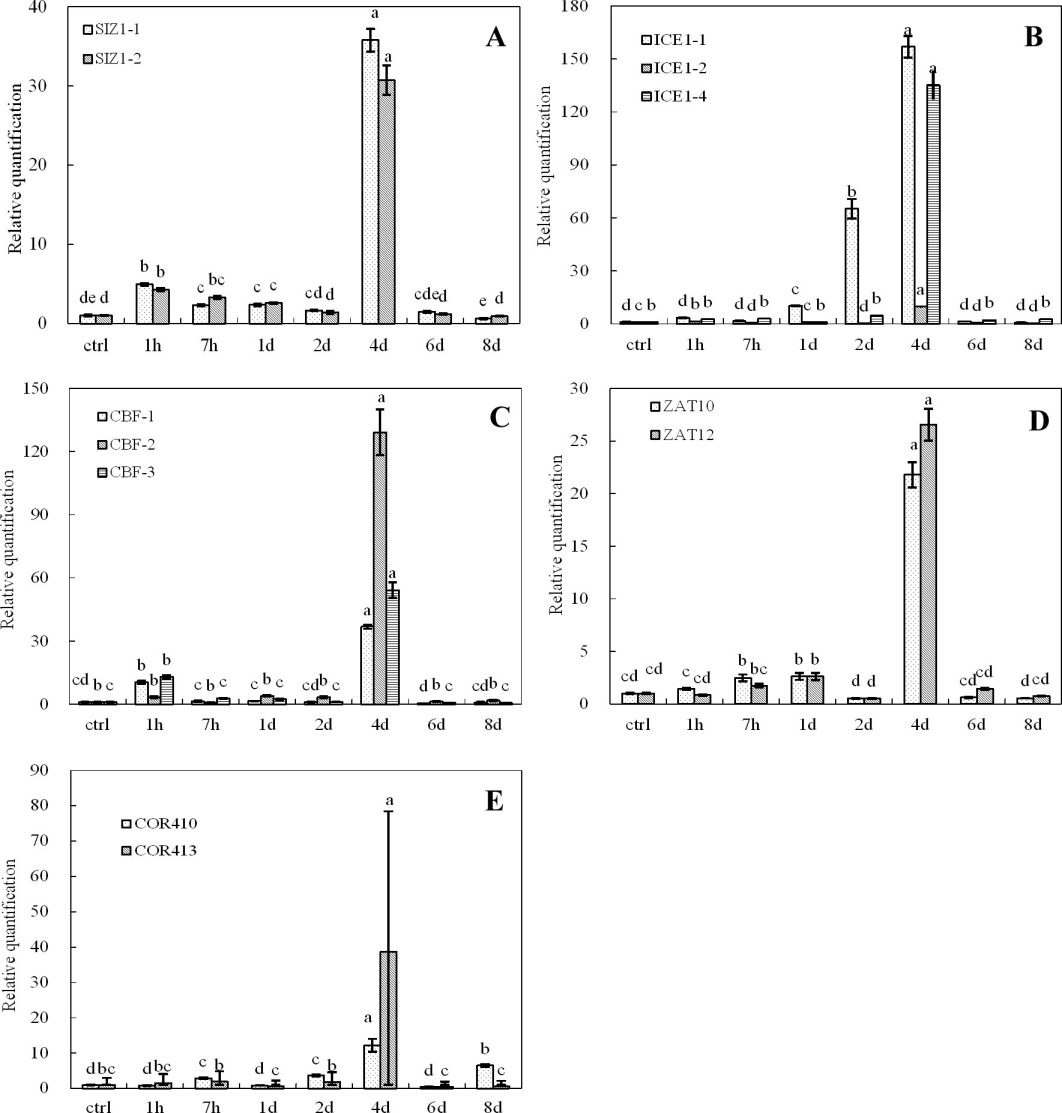
Expression of cold-inducible genes in oil palm under cold stress (12°C). A. *SIZ1*-1 and *SIZ1*-2; B. *ICE1*-1, *ICE1*-2 and *ICE1*-4; C. *CBF1*.*CBF2* and *CBF3*; D. *ZAT10* and *ZAT12*; E. *COR410* and *COR413*. Different lower case letters (a, b, c, d, e) above the bars indicates significant differences between genes expression at different time point (*p* <0.05), correspondingly. a is significantly higher than b, c and d, b is significantly higher than c and d and so on. (on x-axis ctrl = control, h = hour, d = day).

The expression of cold-inducible genes (*COR410*, *COR413*, *CBF1*, *CBF2*, *CBF3*, *ICE1-1*, *ICE1-2*, *ICE1-4*, *SIZ1-1*, *SIZ1-2*, *ZAT10*, *ZAT12*) was examined at low temperatures (4°C, 8°C and 12°C) at different time points (1 h, 7 h, 1 d, 2 d, 4 d, 6 d, 8 d). A control experiment was carried out in parallel at 26°C (Fig 1A–1E, Fig 2A–2E and Fig 3A–3E). The data from real-time qRT-PCR revealed low induction of the majority of genes at very early stages (1 h) of cold stress (4°C) compared to the control treatment. However, the expression levels of all tested genes sharply increased after the day one of cold stress and reached a maximum level on the 4th day. The expression of the genes returned to the basal level on the 8^th^ day of cold treatment (Fig 1A–1E, Fig 2A–2E and Fig 3A–3E). In contrast, the expression of the stress-related genes under 12°C treatment peaked on the 4^th^ day and returned to the basal level on the 6^th^ day (Fig 3A–3E). Overall, the expression of *ICE1-1*, *ZAT10*, *ICE1-4*, *SIZ1-1* and *SIZ1-2* was intensively induced on the 2^nd^ or 4^th^ day of cold treatment (Fig 1A–1E, Fig 2A–2E and Fig 3A–3E). Subsequently, the expression of almost all genes returned to normal after the 6th day of cold treatment.

### Fluctuations in free proline contents under cold stress

**Fig 4:**
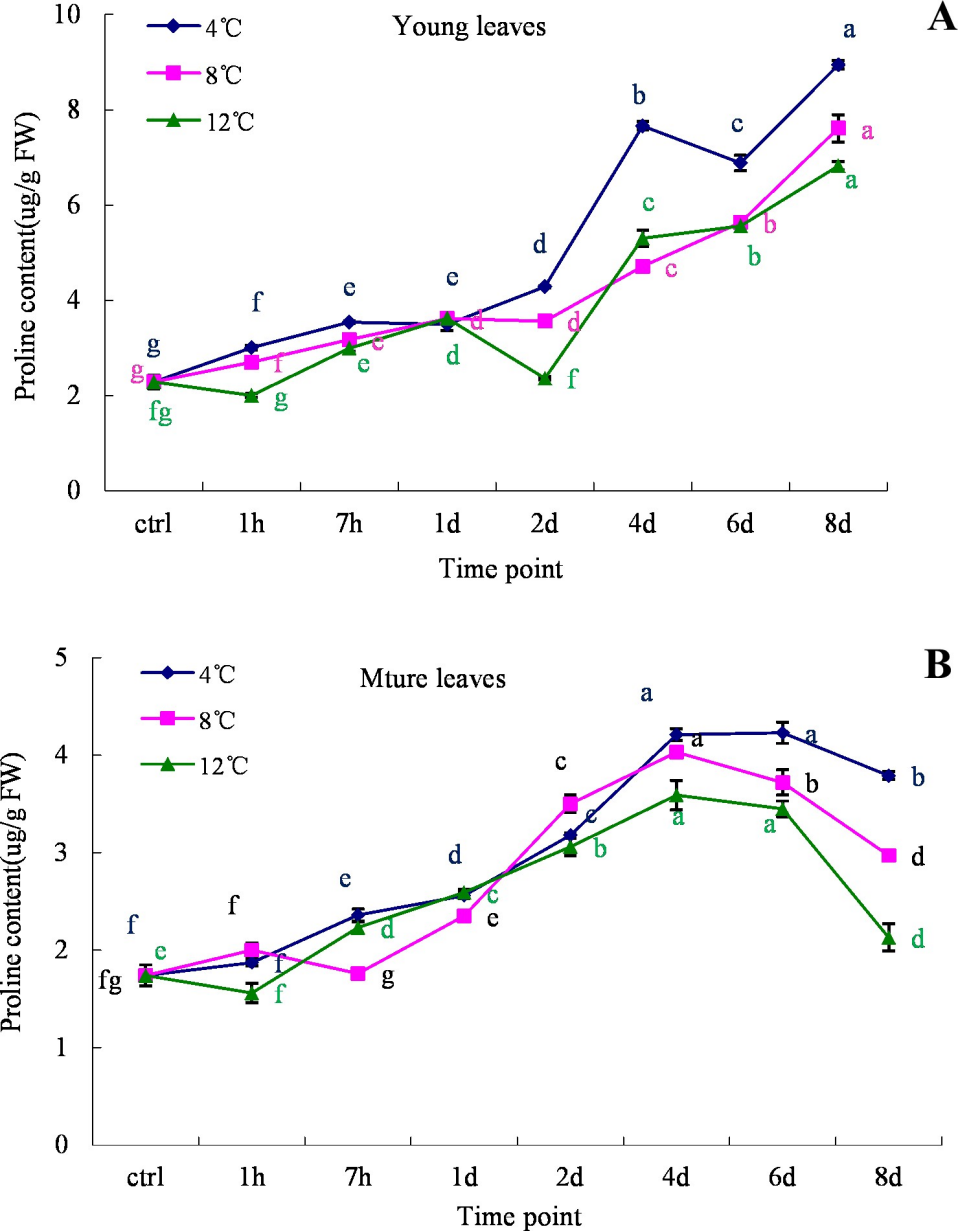
Proline content analysis in young and mature leaves of oil palm under cold stress (4°C, 8°C and 12°C). A. young leaves; B. mature leaves. Different lower case letters (a, b, c, d, e, f) above the bars indicates significant differences between genes expression at different time point (*p* <0.05), correspondingly. a is significantly higher than b, c and d, b is significantly higher than c and d and so on. (on x-axis ctrl = control, h = hour, d = day).

The effect of low temperature on proline contents in relation to induced gene expression was investigated. An increased proline content contributes to the improvement of cold resistance in plants. An increase in proline content was observed in both young and old leaves of oil palm when exposed to cold conditions. In comparison to the control treatment, the proline contents in oil palm leaves under various cold treatments were noticeably higher (Fig 4A & 4B). The proline contents of young leaf samples gradually increased during the cold treatments and reached maximum values of 8.94, 27.61, and 6.82 μg/g FW at 4°C, 8°C, and 12°C, respectively (Fig 4A). However, proline contents in older leaf samples first increased to a maximum value within 4 days of cold treatment and then decreased on the 8^th^ day (Fig 4B). The recorded increase in the proline contents of the young leaves was 5x, whereas that in old leaves was 3x compared to the control (Fig 4A & 4B).

### Changes in relative electrolyte leakage under cold stress

**Fig 5:**
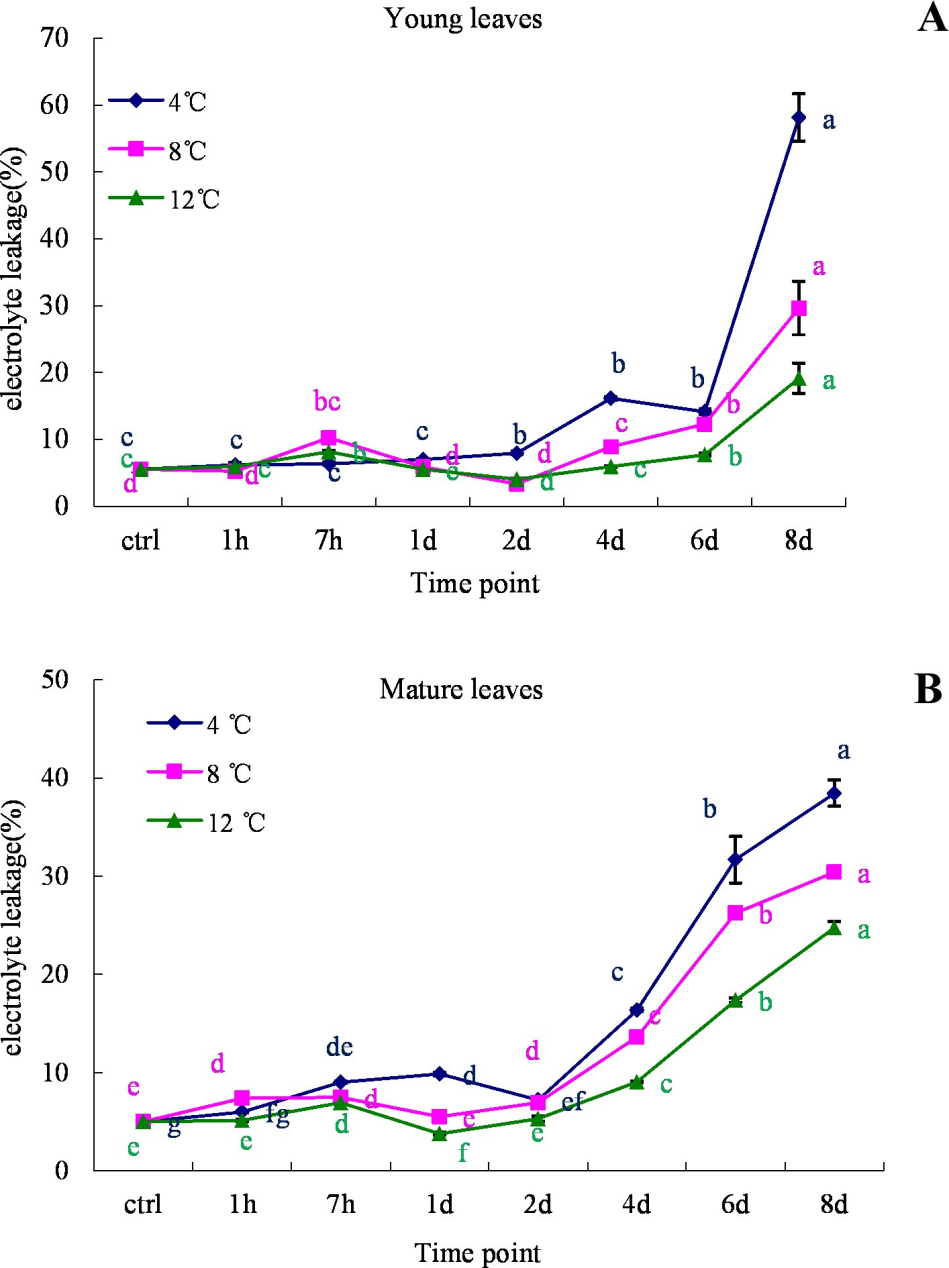
Electrolyte leakage analysis in young and mature leaves of oil palm under cold stress (4°C, 8°C and 12°C). A.young leaves; B. mature leaves. Different lower case letters (a, b, c, d, e) above the bars indicates significant differences between genes expression at different time point (*p* <0.05), correspondingly. a is significantly higher than b, c and d, b is significantly higher than c and d and so on. (on x-axis ctrl = control, h = hour, d = day).

To investigate electrolyte leakage (EL) during cold stress conditions, an experiment was designed to observe the levels of EL in oil palm leaf samples at 4°C, 8°C and 12°C. The results showed that the EL of oil palm leaves was greatly increased until the final time point of the cold treatments (Fig 5A & 5B). The average electrolyte leakage in both young and mature leaves under control conditions was 5%, but cold stress induced electrolyte leakage. During cold stress, the relative electrolyte leakage first gradually increased and then increased more rapidly at the later stages of the incubation period. Electrolyte leakage in both young and mature leaves was significantly higher at 4°C than at 8°C and 12°C. On the 8^th^ day of cold stress, the recorded relative electrolyte leakage in young leaves was 58%, 29% and 19% at 4°C, 8°C and 12°C, respectively (Fig 5A), while in mature leaves, the corresponding electrolyte leakage was 38%, 30% and 24% (Fig 5B).

### Variations in Malondialdehyde (MDA) contents of leaves under cold stress

**Fig 6:**
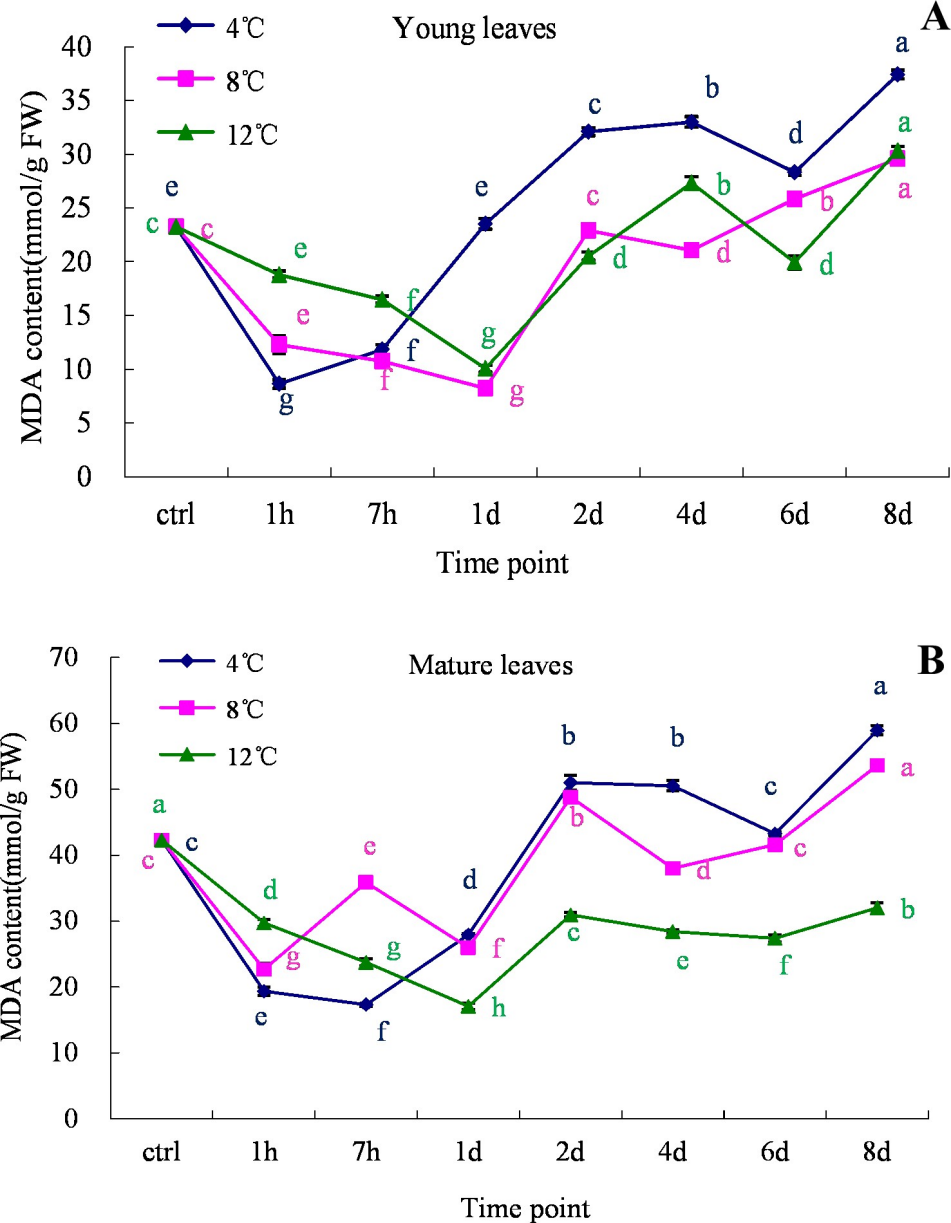
MDA analysis in young and mature leaves of oil palm under cold stress (4°C, 8°C and 12°C). A. young leaves; B. mature leaves. Different lower case letters (a, b, c, d, e) above the bars indicates significant differences between genes expression at different time point (*p* <0.05), correspondingly. a is significantly higher than b, c and d, b is significantly higher than c and d and so on. (on x-axis ctrl = control, h = hour, d = day).

As MDA is one of the indicators of damaged cells, we investigated the effects of cold stress on the MDA contents of oil palm leaf samples. The results shown in Fig 6A & 6B reveal that the MDA contents of both young and mature leaves decreased during the 1^st^ day of the cold treatments (4°C, 8°C and 12°C). Moreover, the MDA concentrations in both young and mature leaves increased after the 1^st^ day of incubation in all the tested treatments. On the 8^th^ day of the cold treatments (4°C, 8°C and 12°C), the MDA contents reached significantly higher levels in young leaves (Fig 6A). Similar observations were recorded in old leaves at 4°C and 8°C, whereas at 12°C, the recorded MDA contents were even lower than in the control treatment (Fig 6B).

### Effect of cold stress on antioxidant enzyme activity

**Fig 7:**
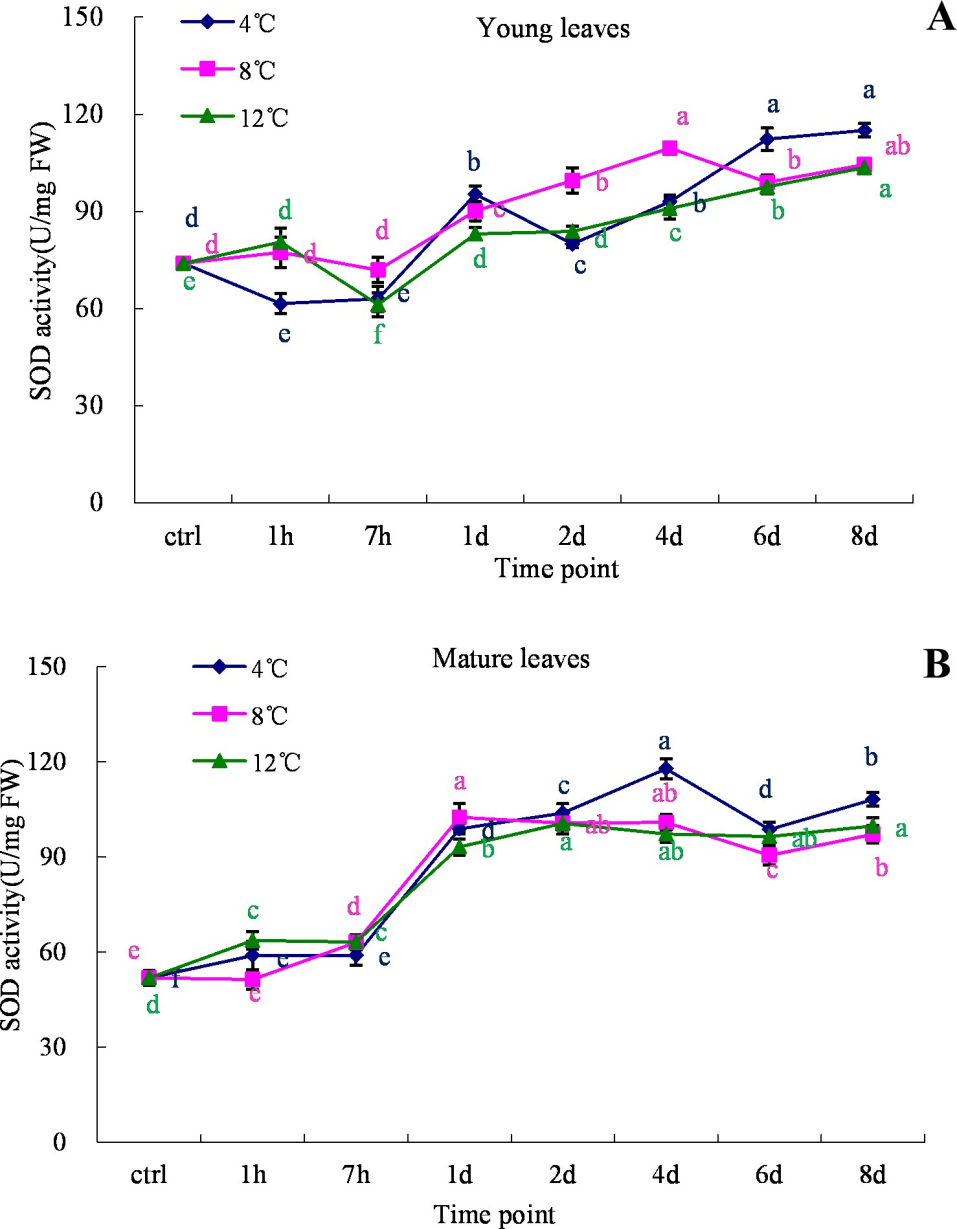
SOD analysis in young and mature leaves of oil palm under cold stress (4°C, 8°C and 12°C). A. young leaves; B. mature leaves. Different lower case letters (a, b, c, d, e) above the bars indicates significant differences between genes expression at different time point (*p* <0.05), correspondingly. a is significantly higher than b, c and d, b is significantly higher than c and d and so on. (on x-axis ctrl = control, h = hour, d = day).

Under stress conditions, the enzymatic system of a living organism is prone to changes. In the present study, we measured the activity of the stress-related enzyme superoxide dismutase under normal and cold stress conditions. Compared to the control condition, SOD activity was higher in both young and mature leaves under the cold stress treatments (4°C, 8°C and 12°C) (Fig 7A & 7B). In the young leaf samples, SOD activity started to decrease in the first 7 h at all treatment temperatures, whereas a gradual increase in SOD activity was noticed thereafter (Fig 7A). On the other hand, no decrease in SOD activity was observed in the mature leaves at any tested temperature (Fig 7B). The activity of SOD in mature leaves treated at 4°C slowly increased during the initial 7 h, after which the rate of increase quickened during 1^st^ to 4^th^ day of incubation and steadied thereafter. In contrast, the changes in SOD activity in mature leaves at 8°C and 12°C were abrupt from 7 h to 1 day and steadied thereafter (Fig 7B).

### Changes in sucrose contents under cold stress

**Fig 8:**
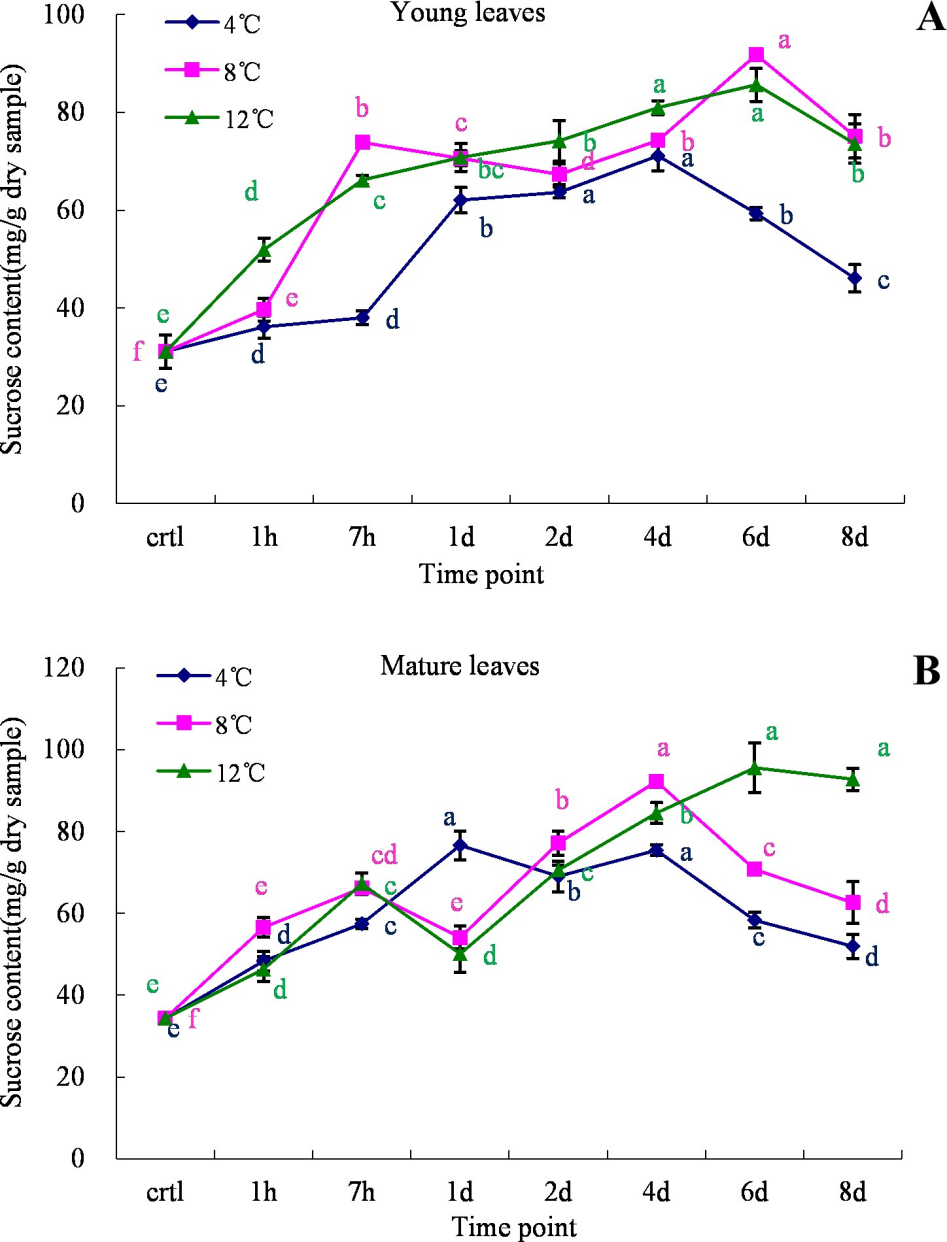
Sucrose content analysis in young and mature leaves of oil palm under cold stress (4°C, 8°C and 12°C). A. young leaves; B. mature leaves. Different lower case letters (a, b, c, d, e) above the bars indicates significant differences between genes expression at different time point (*p* <0.05), correspondingly. a is significantly higher than b, c and d, b is significantly higher than c and d and so on. (on x-axis ctrl = control, h = hour, d = day).

Fig 8A & 8B show that the sugar contents of both young and mature leaves were markedly higher under cold stress conditions. Young leaves under control conditions contained 31.05 mg/g of sucrose, which increased to 71.13 mg/g of sucrose under 4°C cold stress. Furthermore, a rapid increase was observed during the first 7 h of incubation, which then evened off, and a decrease was observed in the later stages of the treatment. Similar trends were observed at 8°C and 12°C, yet the effect was weaker compared to the 4°C treatment (Fig 8A). In the older leaves, the maximum accumulation of sucrose was observed at different time points (Fig 8B). At 4°C and 8°C, the greatest accumulation of sucrose was observed on the 4^th^ day of incubation, whereas in the 12°C treatment, the maximum increase in sucrose was observed on the 6^th^ day of cold stress.

### Correlation of CBFs with other cold-related genes

The *CBF1* and *CBF3* genes showed significant or highly significant positive correlations with *COR410*, *COR413*, *ICE1-1*, *ICE1-2*, *ICE1-4*, *SIZ1-1*, *ZAT10* and *ZAT12* under the 4°C treatment. Similarly, the *CBF1*, *CBF2* and *CBF3* genes showed highly significant positive correlations with all other genes under the 12°C treatment. On the other hand, the *CBF1* and *CBF3* genes showed significant or highly significant positive correlations with *COR413*, *ICE1-1*, *ICE1-4* and *ZAT12* under the 8°C treatment (Table 1). The correlation coefficients between *CBF1* and *COR410*, *COR413*, *ICE1-1*, *ICE1-2*, *ICE1-4*, *SIZ1-1* and *ZAT10* were 0.88 (P<0.01), 0.92 (P<0.01), 0.98 (P<0.01), 0.76 (P<0.05), 0.98 (P<0.01), 0.86 (P<0.05), 0.94 (P<0.01) and 0.79 (P<0.05), respectively. Likewise, the correlation coefficients between *CBF3* and *COR410*, *COR413*, *ICE1-1*, *ICE1-2*, *ICE1-4*, *SIZ1-1* and *ZAT10* were 0.88 (P<0.01), 0.86 (P<0.05), 0.97 (P<0.01), 0.89 (P<0.01), 0.96 (P<0.01), 0.82 (P<0.05) and 0.91 (P<0.01), respectively, at 4°C. *CBF2* showed no significant correlation with other genes under 4°C treatments, but a significant correlation was observed with *COR410*, *ICE1-1* and *ICE1-4* 8°C (Table 1).

**Table 1:** Correlation analysis of CBFs with other cold-related genes in oil palm under cold stress (4°C, 8°C and 12°C).

		COR410	COR413	ICE1-1	ICE1-2	ICE1-4	SIZ1-1	SIZ1-2	ZAT10	ZAT12
	CBF1	0.88**	0.92**	0.98**	0.76*	0.98**	0.86*	0.73	0.94**	0.79*
4°C	CBF2	0.28	0.43	0.54	0.46	0.51	0.55	0.64	0.51	0.58
	CBF3	0.88**	0.86*	0.97**	0.89**	0.96**	0.82*	0.69	0.91**	0.75
	CBF1	0.66	0.85*	0.81*	-0.07	0.85*	0.74	0.67	0.66	0.76*
8°C	CBF2	0.83*	0.56	0.84*	-0.56	0.65	0.58	0.86*	0.44	0.58
	CBF3	0.63	0.99**	0.90**	0	0.98**	0.72	0.64	0.66	0.84*
	CBF1	0.91**	0.98**	0.88**	0.99**	0.98**	0.99**	0.99**	0.97**	0.97**
12°C	CBF2	0.95**	0.99**	0.93**	0.99**	0.99**	0.99**	0.99**	0.99**	0.99**
	CBF3	0.91**	0.97**	0.88**	0.98**	0.97**	0.99**	0.985**	0.97**	0.96**

*Correlation is significant at P<0.05 level (2-tailed)

**Correlation is significant at P<0.01 level (2-tailed)

### Correlation between physiological and biochemical indicators

Table 2 shows a significant positive correlation between electrolyte leakage and proline content (0.99; P<0.01) at 4°C. The correlation coefficients between sucrose and proline at 4°C, 8°C and 12°C were 0.76 (P<0.05), 0.87 (P<0.05) and 0.79 (P<0.05), respectively. The correlation coefficient between SOD and proline at 8°C was 0.79 (P<0.05). A nonsignificant correlation was observed between MDA and other physiological indices (Table 2). The results indicated that proline is a sensitive physiological indicator of oil palm subjected to cold stress.

**Table 2:** Correlation analysis between physiological and biochemical indicators in oil palm under cold stress (4°C, 8°C and 12°C).

		Electrolyte leakage	Proline	MAD	Sucrose	SOD
4°C	Electrolyte leakage	1.00	0.99**	0.28	0.72	0.73
	Proline		1.00	0.29	0.76*	0.71
	MAD			1.00	0.60	0.63
	Sucrose				1.00	0.73
	SOD					1.00
8°C	Electrolyte leakage	1.00	0.67	0.27	0.66	0.14
	Proline		1.00	0.57	0.87*	0.79*
	MAD			1.00	0.24	0.68
	Sucrose				1.00	0.61
	SOD					1.00
12°C	Electrolyte leakage	1.00	-0.03	-0.21	0.01	-0.62
	Proline		1.00	0.23	0.79*	0.67
	MAD			1.00	-0.06	0.26
	Sucrose				1.00	0.57
	SOD					1.00

*Correlation is significant at P<0.05 level (2-tailed)

**Correlation is significant at P<0.01 level (2-tailed)

### Correlation between physiological indicators and cold-related genes

The correlation analysis between physiological indices and cold tolerance genes is presented in Table 3. At 4°C, significant positive correlations between sucrose and *CBF1* (0.68; P <0.05), *CBF2* (0.91; P <0.01) and *SIZI-2* (0.74, P <0.05) were noted. Moreover, a significant correlation was established between SOD and *ICE1-2* (-0.90; P <0.01) at 8°C. The correlation between the various physiological indices and gene expression at 12°C was nonsignificant. However, conductivity was negatively correlated with almost all genes (Table 3).

**Table 3:** Correlation Analysis of Physiological Indicators and Cold-Related Genes in Oil Palm under Cold Stress (4°C, 8°C and 12°C).

Gene	Temperature	Electrolyte leakage	Proline	MAD	Sucrose	SOD
**COR410**	4°C	-0.23	0.22	-0.31	0.39	-0.15
	8°C	-0.18	0.02	0.32	0.26	0.47
	12°C	0.17	0.51	0.67	0.41	0.33
**COR413**	4°C	-0.16	0.32	-0.24	0.50	0.04
	8°C	-0.34	-0.20	-0.04	0.05	0.14
	12°C	-0.23	0.29	0.41	0.33	0.16
**CBF1**	4°C	0.10	0.56	0.11	0.68*	0.37
	8°C	-0.27	-0.03	0.15	0.37	0.27
	12°C	-0.24	0.22	0.38	0.49	0.16
**CBF2**	4°C	-0.12	0.41	0.53	0.91*	0.55
	8°C	0.56	0.73	0.87	0.41	0.85
	12°C	-0.23	0.32	0.41	0.35	0.20
**CBF3**	4°C	-0.02	0.49	0.00	0.63	0.26
	8°C	-0.28	-0.12	0.01	0.17	0.21
	12°C	-0.24	0.20	0.36	0.27	0.14
**ICE1-1**	4°C	0.01	0.49	-0.02	0.59	0.21
	8°C	-0.34	-0.09	0.19	0.12	0.44
	12°C	-0.38	0.18	0.36	0.37	0.18
**ICE1-2**	4°C	0.38	0.76*	0.13	0.39	0.41
	8°C	-0.46	-0.76*	-0.59	-0.51	-0.90**
	12°C	-0.26	0.25	0.37	0.27	0.15
**ICE1-4**	4°C	0.03	0.49	-0.02	0.59	0.20
	8°C	-0.33	-0.13	0.05	0.08	0.26
	12°C	-0.22	0.32	0.42	0.35	0.20
**SIZ1-1**	4°C	-0.04	0.38	0.17	0.68	0.08
	8°C	-0.27	-0.17	0.01	0.28	0.13
	12°C	-0.25	0.26	0.36	0.33	0.16
**SIZ1-2**	4°C	-0.09	0.35	0.32	0.74*	0.09
	8°C	-0.22	0.01	0.32	0.27	0.49
	12°C	-0.22	0.26	0.35	0.33	0.13
**ZAT10**	4°C	-0.02	0.42	0.05	0.63	0.11
	8°C	-0.23	-0.19	-0.09	0.26	0.03
	12°C	-0.23	0.29	0.34	0.34	0.13
**ZAT12**	4°C	-0.10	0.33	0.22	0.70	0.04
	8°C	-0.31	-0.22	-0.04	0.16	0.11
	12°C	-0.23	0.32	0.36	0.36	0.18

*Correlation is significant at P<0.05 level (2-tailed)

**Correlation is significant at P<0.01 level (2-tailed)

## Discussion

Cold stress can be classified as chilling (0–15°C) and freezing (<0°C) stresses. Generally, plants originating from temperate regions, such as spinach and *Arabidopsis*, exhibit a variable degree of chilling tolerance and can increase their freezing tolerance during exposure to chilling and non-freezing temperatures. This process is known as cold acclimation [14]. On the other hand, plants of tropical and subtropical origins are sensitive to chilling stress and lack the cold acclimation mechanism [27]. Oil palm is a tropical crop with the highest oil yield in the world. The most suitable temperatures for oil palm are above 25°C and below 28°C. At such temperatures, oil palms grow well and accumulate high oil contents. In contrast, at lower temperatures (below 15° C), oil palm struggles to grow and flourish, and thus, its oil contents decrease drastically. Indeed, there are certain genes or transcription factors in oil palm and other species that are activated in response to cold. Information regarding the expression and transformation of such genes will provide us with the knowledge to develop cold-tolerant cultivars. In an attempt to understand the molecular mechanism associated with cold stress in *Elaeis guineensis*, Lei et al. [5] applied Illumina sequencing technology and obtained 51,452 transcripts. In their study, they aligned the expressed sequences by using various databases and the results revealed the induction of many genes that are involved in DNA repair, amino acid metabolism and biosynthesis of cold tolerance molecules. Among them, a sizable number of genes containing CBF (transcription factor) domains were either induced or repressed. The CBF (C-repeat binding factor) cascade involves a series of transcription factors, including *ICE1*, *HOS1*, *MYB15*, *SIZ1* and *ZAT10*, that transmit cold signals and initiate immediate responses to cold stress [28]. In the current study, the expression levels of *ICE1-1*, *ZAT10*, *ICE1-4*, *SIZ1*, *SIZ12* and *SIZ1-2* were also highly induced in response to cold stress. Our results confirmed that similar to other species, putative *ICE1* and *CBF* orthologs were strongly induced in *Elaeis guineensis* under cold stress. In model plant species, the CBF-mediated signal transduction pathway has been shown to play a central role in cold tolerance. It is possible that the CBF-mediated pathway performs a similar role when oil palm is exposed to cold stress. COR413 is a cold-regulated plasma membrane protein plays an important role in conferring freezing tolerance in *Arabidopsis* [29]. In our study, *COR413* expressions show different pattern when suffer to varying degrees of cold treatment. The expression of COR413 observed two peaks at 1h and 4d for 4°C treatment and 2h and 2d for 8°C treatment. However only one peak was show up for 12°C treatment (Figs 1E, 2E and 3E). The *COR* genes expression was regulated by CBF-dependent and CBF-independent pathways, therefore *CBF* and *COR413* gene expression may be differentially (Figs 1C, 2C and 3C). Furthermore, oil palm is a tropical crop the temperature at 4°C can be regards as freezing condition in comparison with temperate crop which may alter the cold response network for this crop. As *COR413* is a membrane protein and may involve in the early cold signaling at freezing condition [30], so its expression may induce in the early cold stress response (Figs 1E and 2E).

*CBF* genes are key players in cold response and adaptation in plants. *CBF1* and *CBF3* are early cold response genes which may involve in the first round of cold acclimation by activating a set of cold responsive genes under cold stress [31, 32]. It has been shown that *CBF1–3* expression is not only responsive to low temperatures, but is also gated by the circadian clock. The cold-induced up regulation of *CBF* genes also depends on the time of day[8]. The three *CBFs* do not have fully overlapping functions. *CBF1* and *CBF3* play a different role than *CBF2* in both constitutive freezing tolerance and cold acclimation [16]. The *CBF2* protein is involved in feedback regulation of *CBF1* and *CBF3* expression during cold acclimation[33]. In our study, *CBF1* and 3 expression were induced at its early stage than come down to the ground level may duo to the feedback and circadian clock effect. After prolong cold stress *ICE1*expression was increase at day2 and reach the maximum at day4 (Fig 3B) which induce another round of *CBF1* and *CBF3* in combine with *CBF2*. The expression level of *CBF1* and *CBF3* were lower than *CBF2* may duo to the feedback inhibition of CBF1 and CBF3 by CBF2 (Fig 3C)[33]. The Expression of *CBFs* were also clear link with *ICE1*, *SIZ1*, *ZAT10*, *COR413*, *and ZAT12* expression (Fig 3A, 3B, 3C, 3D and 3E and Table 1), in particularly when then cold treatment were done at 12° C which will be an effective low temperature for oil palm and other tropical corps.

The activation of the CBF cold response pathway result in the induction of a set of COR enzymes and lead to dramatic changes in metabolic reactions. Osmotic adjustment is one of the major ways for plants to adapt to adverse environmental stresses. Proline is an organic osmolyte that accumulates in various plant species during stress conditions [29, 34] stabilizing membranes thereby preventing electrolyte leakage and ROS scavenging [29]. On the other hand, sucrose is a disaccharide that can serve as an energy reservoir for various metabolic processes. Plants can recover from stress conditions via the expression of certain genes during stress [35]. Under cold stress, a set of genes can then trigger the accumulation of proline and sugar, which are known to send signals to regulate mitochondrial functions. In addition, proline can also help stabilize cellular structure and maintain osmotic balance, affecting cell proliferation/cell death [36]. In the current study, as excepted the proline content is increase over the time during cold stress and slight decrease at 8 day for mature leave (Fig 4). A high degree of correlation with electrolyte leakage was observed at 4° C and SOD at 8° C (Table 2) which may be an indication that cell trigger the accumulation of proline try to prevent electrolyte leakage and scavenging ROS under cold stress. Soluble sugars have been confirmed to play an important role during cold acclimation to protect plant cells from damage by serving as osmoprotectants, nutrients as well as interacting with the lipid bilayer. Sucrose, one of the soluble sugars was accumulated in spinach leave[37], wheat[38, 39] and many other crop[40] during the seasonal cold acclimation with high sucrose synthase activity. The sucrose was also accumulated in oil palm leave under cold stress for both young and mature (Fig 8) and it is correlated with the increasing proline content at 4°C, 8°C and 12°C (Table 2). A clear link with the CBF1 and CBF2 genes expression at 4°C were also observed in this study (Table 3).The accumulation of proline and sugar also play a role in helping the plant recover from cold stress when environment condition was reverse. Similarly, an increase in proline and sugar contents has been reported in *ZmMKK4*, *OsMYB2*, *OsMYB4* and *OsMYB3R-2* overexpressing transgenic plants under cold stress [35, 41–43]. Improvement of cold tolerance of crop plants via engineering proline metabolism is an existing possibility and should be explored more extensively.

Under stress conditions, the generation and accumulation of ROS (hydroxyl radicals, superoxide, hydrogen peroxides) are highly damaging to macromolecules and cellular integrity, ultimately leading to cell death [44, 45]. The evaluating of ROS trigger the cellular antioxidant system which is an important pathway of cell physiology and biochemistry (Theocharis et al., 2012). Plants defend themselves under such threats by activating the ROS-scavenging enzyme system. The effective ROS-scavenging enzyme system involves peroxidase (POD), superoxide dismutase (SOD) and catalase (CAT) [46, 47]. SODs can catalyze the dismutation of superoxides [48], which might be the reason that SOD activity under stress conditions can help increase the tolerance level of a plant. SOD activity was higher in both young and mature leaves of oil palm under cold stress over 8days during the experiments (Fig 7). The elevation in SOD activity reflects the activation of the defense mechanism against cold stress in oil palm. Similarly, high SOD activity has been reported by various scientists in different plant tissues during cold stress [35, 49–51]. These findings confirm that plants attempt to resist threats by activating their own defense system.

Malondialdehyde (MDA) is a product of the peroxidation of unsaturated fatty acids that might play a vital role in damaging the cell membrane. Cold stress often causes damage to cells, which in turn results in the deterioration of membrane transport systems and cellular metabolism. Such damage can be indicated by the analysis of MDA. High accumulation of MDA has been observed in various species subjected to cold stress [35, 50, 51], which indicates the link between cold stress and membrane integrity. In planta, cell membranes are considered to be the principal site of injury at a time of stress [52, 53]. Therefore, the damage due to cold stress might lead to high electrolyte leakage and accumulation of MDA. In the present study, the levels of MDA increased in both young and old leaves of oil palm under cold stress conditions. The results also show that the lower temperature the high MDA accumulation (Fig 6A and 6B) after one day cold stress. Indeed, cell membranes are mainly composed of lipid bi-layers, and the peroxidation of lipids results in MDA as a final product [54]. The accumulated MDA can also aid in ROS scavenging, however the high levels of MDA is toxic to plants [49].

Cold stress triggers the formation of ROS and peroxidation of unsaturated fatty acids will alter the membrane structure which is often damaged due to cold stress. Electrolyte leakage is a hallmark of stress response in intact plant cells and used as a test for the stress-induced injury of plant tissues and ‘a measure’ of plant stress tolerance [55, 56]. Our study show the electrolyte leakage of functional and young leaves increased rapidly from the 2^nd^ day to 8^th^ day of cold treatment (Fig 5), which indicate that the cell membranes might have been damaged under low temperature in oil palm. Liu et al., [49] also demonstrated that relative electrolyte leakage was maximal at low temperatures (cold stress) and progressively increased during the prolonged exposure of the seedlings to cold stress. The rate at which electrolytes leak from the cell membrane parallels the damage to cell membrane integrity and functionality [57].

As plants live in open habitats, they suffer due to changes in the environment. Plants acquire low-temperature tolerance via cold acclimation, which involves an array of physiological and biochemical modifications. The *COR* genes include the low-temperature-induced (LTI), responsive to desiccation (RD), and early dehydration-inducible (ERD) genes. Some of these genes encode key enzymes in osmolyte biosynthesis that increase low-temperature tolerance. Such tolerance can be achieved via the accumulation of cryoprotective proteins, proline and soluble sugars to repair cold-rigidified membranes and stabilize the cellular osmotic potential [28]. Our studies also demonstrated the accumulation of osmolytes (proline, MAD and sucrose) during cold stress (Figs 4, 6 and 8). SOD activity increased with the production of ROS under cold stress (Fig 6). CBFs are transcription factors that are critical for cold acclimation in higher plants [58, 59]. When plants are exposed to nonfreezing low temperatures, *CBF* genes can rapidly induce and activate the downstream target *COR* genes as the *CBF* regulon [59–61]. Here we also showed that *CBFs* (especially *CBF1* and *CBF3*) exhibit co-expression with *COR*, *ICE*, *SIZ* and *ZAT10* and 12 (Table 1). *CBF* expression presented a positive correlation with sucrose content when oil palm was subjected to cold stress (Table 2). In our study, the cold stress at different temperature (4°C, 8°C,12°C) show alter response in term of gene expression and at 12°C the expression of *CBF* genes were clear correlated with all other genes expression in this study (Fig 3A–3E and Table 1). However, at 4°C and 8°C the expression the CBF genes and other *COR* genes give a mix pattern. Since oil palm is a tropical crop the temperature at 4°C can be regards as freezing-like condition in comparison with temperate crop which may alter the cold response network for this crop.

## Conclusions

From this study, we conclude that there is a clear link among the expression of *CBFs* and the other genes examined under cold stress at 12°C, with the upstream regulators *ICE1* and *SIZ1* in particular. *CBF1* and *CBF2* show a positive correlation with sucrose and proline contents under cold stress in oil palm. These results provide necessary information for understanding the mechanism of the stress response and the adaption of oil palm to cold conditions. This study will also help breeders and molecular biologists to evaluate existing germplasms for cold tolerance and/or to develop new stress-resistant cultivars.

## Supporting information

S1 Table The primers used in this study. (DOC)
