# Supplementary material for: Correlation analysis of cold-related gene expression with physiological and biochemical indicators under cold stress in oil palm
Source: PLoS One. 2019 Nov 27;14(11):e0225768. doi: 10.1371/journal.pone.0225768 (PMC6881061; doi:10.1371/journal.pone.0225768)
Supplement: S1 Table — (DOC) [file pone.0225768.s001.doc]

**S1 Table. Primers used in this study**

| **Gene** | forward primer**（5’ to 3’）** | reverse primer**（5’ to 3’）** | **Production length (bp)** |
| --- | --- | --- | --- |
| Egactin | GTTGTCGCTCCACCCG | GCAGGACCACATTCATCATA | 119 |
| COR410 | AGGTGAAGGATAGGGGGTTGT | AGACCGGTGGAGCTTTTCAAG | 152 |
| COR413 | TAAGGGATGGTCCAGTCGGT | GAAAGAGTTCCTGAAGCCGC | 215 |
| CBF1 | CAGCTATGTCGATGGCAGGG | CTCCTCGTCGGCGTACAATA | 214 |
| CBF2 | CCGCCCAACTCCAAACTC | GTCTGCGAGGTGCGTGAG | 132 |
| CBF3 | GGCATACTCGACTGTGTGGT | CCTCGACTTCTTGTTGGGCT | 148 |
| ICE1-1 | AAAGCTTCGAGAACGCTCCA | GGTCACCAACGAATTCTGGC | 166 |
| ICE1-2 | AGGGCTGGACATTCAACAGG | ATCGTACTTGGGAAGCCTGC | 230 |
| ICE1-4 | GAGTTGGGGAGAACGGACAG | GCGGTTTAGAAACGGCGAAT | 215 |
| SIZ1 -1 | AGGAATCAAGAAAAATCGCAA | TCGGTTGGACTGCTGCTC | 198 |
| SIZ1 -2 | CCGTATTTCAACCGCATCAC | GTTTGCCACATAAAGACTACCAT | 210 |
| ZAT10 | GTGGCAAGGCTTTCCCTTCT | CCGGACGTCTTCAATCCTGG | 205 |
| ZAT12 | CGACCGAGAGTTTTGGGCA | GATGGCGGCAGGTTCAGAT | 182 |
